# Intravenous high‐dose interferon with or without maintenance treatment in melanoma at high risk of recurrence: meta‐analysis of three trials

**DOI:** 10.1002/cam4.563

**Published:** 2015-12-08

**Authors:** Agnieszka Malczewski, Andrea Marshall, Miranda J. Payne, Lili Mao, Dimitrios Bafaloukos, Lu Si, Dimitrios Pectasides, George Fountzilas, Jun Guo, Helen Gogas, Mark R. Middleton

**Affiliations:** ^1^ National Institute for Health Research Biomedical Research CentreOxford University Hospitals NHS TrustOxfordUnited Kingdom; ^2^ Warwick Clinical Trials UnitUniversity of WarwickCoventryUnited Kingdom; ^3^ Department of Renal Cancer and MelanomaKey Laboratory of Carcinogenesis and Translational ResearchMinistry of EducationPeking University Cancer Hospital & InstituteBeijingChina; ^4^ Hellenic Co‐operative Oncology GroupAthensGreece

**Keywords:** Adjuvant, interferon, melanoma, meta‐analysis, survival

## Abstract

Resected stage IIB–IIIC malignant melanoma has a poor prognosis with a high risk of relapse and death. Treatment with adjuvant interferon alfa‐2b (IFN‐*α*‐2b) is associated with improved relapse‐free and overall survivals (OS), but the most appropriate dose and duration of treatment are unknown. In this article, we present an individual patient data random effects meta‐analysis of melanoma patients from the U.K., Greek, and Chinese randomized trials. All patients were randomized either to IFN‐*α*‐2b 15–20 MIU/m^2^ IV daily 5 days per week for 4 weeks (IV) or to the same regimen followed by IFN‐*α*‐2b 9–10 MIU/m^2^ administered three times per week for 48 weeks (IV and SC). Allowing for dose interruptions and reductions, an equivalent total dose of IFN‐*α*‐2b was delivered in all three studies. We assessed whether IV was noninferior to IV and SC in terms of relapse‐free survival (RFS) and investigated tumor and patient characteristics that impacted on outcomes. Median follow‐up of 716 stage IIB–IIIC patients was 5.4 years. Noninferiority of IV compared to IV and SC could not be conferred for RFS (hazard ratio [HR] 1.16, 95% confidence interval [CI] 0.89–1.52; noninferior *P* = 0.17). Stage (*P* < 0.0001), site (acral vs. other, *P* < 0.0001), and Breslow thickness (*P* = 0.02) were significant predictors of RFS. The HR for death was 1.13 for IV compared to IV and SC, (95% CI 0.91–1.39). Stage (*P* < 0.0001) and Breslow thickness (*P* = 0.001) were significant independent predictors of OS. The available data suggest that where adjuvant high‐dose interferon is being considered there is no evidence to deviate from the year long regimen described in the Eastern Cooperative Oncology Group and Intergroup studies.

## Introduction

The incidence of malignant melanoma continues to rise with over 230,000 new cases annually worldwide and more than 55,000 deaths estimated in 2012 1. Patients with thick primaries, ulcerated lesions, or regional lymph node metastases have a high risk of relapse with 5‐year mortality rates of 40–80% 2.

To date, interferon alfa‐2b (IFN‐*α*‐2b) is the only therapy that has gained approval in the United States and Europe for the adjuvant treatment of high‐risk resected melanoma. A recent meta‐analysis of 14 randomized controlled trials showed that adjuvant interferon was associated with significantly improved disease‐free survival (*P* < 0.001) and overall survival (OS) (*P* = 0.002) 3. Despite this, the optimal dose and treatment duration remain uncertain. It is also unclear whether certain prognostic subgroups (e.g., patients with clinically positive lymph nodes) would derive greater benefit from particular doses or durations of interferon treatment. There is some evidence to suggest that patients with ulcerated tumors derive most benefit from intermediate doses 4.

A common adjuvant regimen has evolved from the Eastern Cooperative Oncology Group (ECOG) and Intergroup trials 5, 6, 7. The regimen consists of an induction phase of daily intravenous (IV) interferon at 20 MIU/m^2^ per day, which is administered for 5 consecutive days of the 7 for 4 weeks. This is followed by a 48‐week maintenance period, during which subcutaneous interferon is administered at 10 MIU/m^2^ three times per week (SC). In the ECOG/Intergroup 1684, 1690, and 1694 studies 5, 6, 7, dose delays or reductions for toxicity were required in 37%, 44%, and 28% of patients, respectively, during the induction phase and in 36%, 52%, and 37% during maintenance. The survival curves in ECOG1684 5 separated well before a year, the planned duration of treatment, raising the possibility that the early very high dose component of the regimen drives its efficacy. Three trials have been conducted to examine this question, which have been published elsewhere 8, 9, 10. We present an individual patient data meta‐analysis of these study populations. We aimed to assess whether a shorter duration of high‐dose interferon (HDI) treatment was noninferior to longer treatment in terms of relapse‐free survival, and also to explore patient and tumor characteristics that might impact on outcome and interact with the effects of the drug.

## Methods

Studies eligible for inclusion were those comparing the intravenous month of the ECOG HDI regimen (IV) with the full year of therapy (IV and SC) from which individual patient data were available. Trials databases (clinicaltrials.gov, ISRCTN) and the literature (Pubmed, Cochrane database) were searched using the terms Interferon AND Melanoma AND Adjuvant to identify potential studies. Trials comparing a year of interferon therapy to repeated administration of the IV month were excluded. Three studies were identified from the United Kingdom (U.K.), Greece and China, respectively.

The U.K. phase II trial compared the intravenous month of the ECOG HDI regimen (IV) with the full year of therapy (IV and SC) at the doses described above 8. In the Greek phase III trial and the Chinese phase II trial, the IV dose was 15 MIU/m^2^ per day and, for those patients receiving a year of treatment, the SC doses used were 10 and 9 MIU three times a week, respectively 9, 10. Data were obtained from all three trials on survival and relapse information, trial arm, and the characteristics of gender, age, stage of disease, site of disease, Breslow thickness, and ulceration. Relapse‐free survival (RFS) was calculated as the time from entry into the trial until the date of first relapse or death from any cause. Patients were censored at the date last seen if alive and relapse free. OS was calculated as the time from date of entry in the trial until date of death from any cause.

All analyses were carried out on an intention‐to‐treat basis, including all patients according to their assigned treatment allocation, irrespective of whether they received the treatment or were previously excluded from analyses by the investigators. Due to missing data, multiple imputations were performed using fully conditional specification method 11 within SAS (version 9.3; Cary, NC) statistical package with 10 imputations and the results combined using Rubin's rules 12.

A one‐stage random effects meta‐analysis was performed and the results presented as a hazard ratio (HR) plot. The between study heterogeneity was assessed using the Cochran's *Q* statistic 13 and the *I* ^2^ statistic. Due to the low power of any heterogeneity test, a one‐stage random effects Cox regression model was undertaken 14 to assess the treatment effect and treatment–covariate interactions. Multivariable Cox regression models were fitted to investigate the independent predictors of RFS and OS.

With 700 patients, the meta‐analysis had 90% power to determine noninferiority at the 2.5% level with a large 10% noninferiority margin, assuming a 50% RFS at 2 years for the standard IV and SC arm, a recruitment period of 6 years, and a minimum of 2 years follow‐up. Noninferiority of RFS with a 10% margin would be conferred if the 97.5% quantile of the HR is less than 1.32.

## Results

Searching trials and publications databases using the terms Interferon AND Melanoma AND Adjuvant yielded 84 and 205 reports, respectively. The citations in published reports of adjuvant interferon trials were also searched for references to additional studies. From these we identified three studies meeting our criteria, and two additional studies in which a year of treatment was compared to repeated administration of the IV month of the drug.

Data from 716 patients in the three trials were analyzed (Table 1). There were differences in the study populations of the three trials. The Chinese population was designed to recruit only patients with acral melanoma, to explore HDI in the prevailing histology in that territory. The U.K. population consisted of a higher proportion with stage III disease (80% vs. 58% or 57%), the majority of which was clinically detectable. More Chinese patients had ulcerated tumors, but median age and gender distribution were similar in the three cohorts.

**Table 1:** Patient characteristics, events, and length of follow‐up by cohort

Cohort	I (U.K.)	II (China)	III (Greece)	Total
*Characteristic*				
Sample size	194	158	364	716
Trial arm				
IV	96 (49%)	79 (50%)	182 (50%)	357 (50%)
IV and SC	98 (51%)	79 (50%)	182 (50%)	359 (50%)
Gender				
Female	87 (45%)	74 (47%)	180 (49%)	341 (48%)
Male	107 (55%)	84 (53%)	184 (51%)	375 (52%)
Stage of disease				
II	38 (20%)	67 (42%)	110 (30%)	215 (30%)
III	156 (80%)	91 (58%)	207 (57%)	454 (63%)
Not recorded	0	0	47 (13%)	47 (7%)
Breslow thickness				
≤1 mm	24 (12%)	5 (3%)	13 (3%)	42 (6%)
>1–2 mm	39 (20%)	15 (9%)	37 (10%)	91 (13%)
>2–4 mm	53 (27%)	81 (51%)	101 (28%)	235 (33%)
>4 mm	59 (31%)	57 (36%)	163 (45%)	279 (39%)
Unknown	19 (10%)	0	50 (14%)	69 (9%)
Ulceration				
Yes	72 (37%)	104 (66%)	154 (42%)	330 (46%)
No	63 (32%)	54 (34%)	100 (28%)	217 (30%)
Unknown	59 (30%)	0	110 (30%)	169 (24%)
Age (years)				
Median (range)	49 (17–78)	49 (22–76)	53 (19–83)	50 (17–83)
Site of primary tumor				
Acral	13 (7%)	158 (100%)	15 (4%)	186 (26%)
Head and neck	18 (9%)	0	60 (17%)	78 (11%)
Trunk	80 (41%)	0	118 (32%)	198 (27%)
Limbs	75 (39%)	0	138 (38%)	213 (30%)
Other	0	0	14 (4%)	14 (2%)
Unknown	8 (4%)	0	19 (5%)	27 (4%)
Number alive	97 (50%)	70 (44%)	207 (57%)	374 (52%)
Median follow‐up in years (interquartile range)	5.7 (4.7–7.1)	6.2 (5.9–6.4)	4.7 (2.7–6.3)	5.4 (3.5–6.5)
Number of deaths	97 (50%)	88 (56%)	157 (43%)	342 (48%)
Number of relapses	109 (56%)	115 (73%)	217 (60%)	441 (62%)
Number of relapses or deaths	113	118	217	448

IV, trial arm receiving IFN‐*α*‐2b 15–20 MIU/m^2^ IV daily 5 days per week for 4 weeks; IV and SC, trial arm receiving IFN‐*α*‐2b 15–20 MIU/m^2^ IV daily 5 days per week for 4 weeks followed by IFN‐*α*‐2b 9–10 MIU/m^2^ administered three times per week for 48 weeks.

At the time of analysis, the median follow‐up in surviving patients across all trials was 5.4 years (range 0–9.6 years) with 441 (62%) relapses and 342 (48%) deaths (Table 1). The proportion of relapses and deaths and length of follow‐up were similar across all three trials (Table 1).

**Figure 1:**
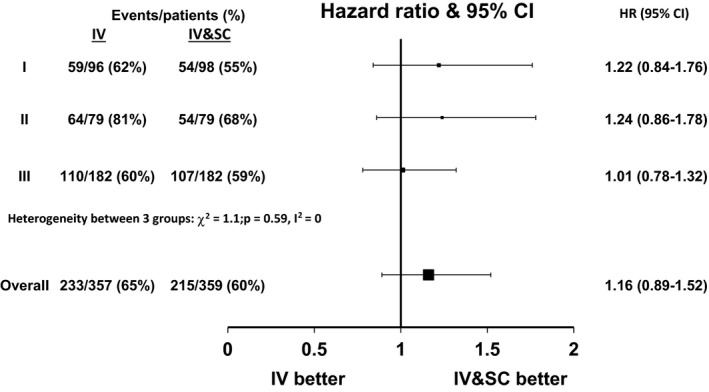
Hazard ratio plot of the treatment effect for relapse‐free survival.

There appeared to be no significant heterogeneity between the cohorts with respect to RFS (*χ* ^2^ = 1.1, *P* = 0.59, *I* ^2^ = 0, Fig. 1). The treatment effect estimate was consistent and nonsignificant across all trials (Table 2). The overall HR for relapse or death was 1.16 for the 4 weeks IV arm compared to the IV and SC arm (95% confidence interval [CI] 0.89–1.52, Fig. 1). Noninferiority of the shorter IV arm could not be conferred (*P* = 0.17). Treatment–covariate interactions were explored in a one‐stage random effects model for RFS, but none were significant (Table 3). In a multivariable one‐stage random effects analysis, stage (III vs. II, HR = 1.82, 95% CI 1.45–2.28, *P* < 0.0001), site (acral vs. other, HR = 1.59, 95% CI 1.28–1.96, *P* < 0.0001), and Breslow thickness (>4 mm vs. ≤4 mm, HR = 1.16, 95% CI 1.02–1.33, *P* = 0.02) were significant independent predictors of RFS (Table 4).

**Table 2:** RFS and OS results from the individual patient data analysis for each cohort

Cohort	Arm	*N*	No. of events	Median in years (95% CI)	% event free at 2 years	HR (95% CI)	*P*‐value
Relapse‐free survival							
I (U.K.)	IV	96	59	2.0 (1.2–3.2)	50	1.22 (0.84–1.76)	0.29
	IV and SC	98	54	2.8 (1.5–a)	54		
II (China)	IV	79	64	1.5 (0.8–2.4)	44	1.24 (0.86–1.78)	0.24
	IV and SC	79	54	1.9 (1.0–2.8)	46		
III (Greece)	IV	182	110	2.1 (1.6–3.7)	52	1.01 (0.78–1.32)	0.93
	IV and SC	182	107	2.3 (1.7–3.6)	52		
OS							
I (U.K.)	IV	96	54	3.5 (2.9–a)	67	1.39 (0.93–2.07)	0.11
	IV and SC	98	43	a(3.7–a)	71		
II (China)	IV	79	50	5.3 (4.2–5.8)	97	1.44 (0.94–2.20)	0.09
	IV and SC	79	38	5.9 (4.9–a)	94		
III (Greece)	IV	182	78	5.4 (4.7–a)	79	0.91 (0.66–1.24)	0.54
	IV and SC	182	79	5.6 (3.7–a)	73		

RFS, relapse‐free survival; OS, overall survival; HR, hazard ratio; IV, trial arm receiving IFN‐*α*‐2b 15–20 MIU/m^2^ IV daily 5 days per week for 4 weeks; IV and SC, Trial arm receiving IFN‐*α*‐2b 15–20 MIU/m^2^ IV daily 5 days per week for 4 weeks followed by IFN‐*α*‐2b 9–10 MIU/m^2^ administered three times per week for 48 weeks.

a Limit not reached.

**Table 3:** Treatment–covariate interactions in a one‐stage random effects model for recurrence‐free survival and overall survival

Covariate	*P*‐value for covariate	*P*‐value for treatment–covariate interaction
Model for relapse‐free survival		
Stage	0.001	0.60
Gender	0.88	0.27
Site of disease	0.03	0.50
Breslow group	0.32	0.69
Ulceration	0.80	0.70
Model for overall survival		
Stage	0.001	0.96
Gender	0.34	0.98
Site of disease	0.64	0.51
Breslow group	0.08	0.56
Ulceration	0.99	0.78

**Table 4:** Results of a multivariable one‐stage random effects models for relapse‐free survival and overall survival

Factor	Hazard ratio	95% confidence interval	*P*‐value
Model for recurrence‐free survival			
Treatment			0.16
IV and SC	1.00		
IV	1.14	0.95–1.40	
Stage			<0.0001
II	1.00		
III	1.82	1.45–2.28	
Site of disease			<0.0001
Other sites	1.00		
Acral	1.59	1.28–1.96	
Ulceration			0.99
Yes	1.00		
No	1.00	0.80–1.25	
Breslow thickness			0.02
≤4 mm	1.00		
>4 mm	1.16	1.02–1.33	
Model for overall survival			
Treatment			0.26
IV and SC	1.00		
IV	1.13	0.91–1.41	
Stage			<0.0001
II	1.00		
III	2.21	1.66–2.90	
Site of disease			0.77
Other sites	1.00		
Acral	1.04	0.82–1.32	
Ulceration			0.88
Yes	1.00		
No	0.99	0.75–1.27	
Breslow thickness			0.001
≤4 mm	1.00		
>4 mm	1.32	1.12–1.56	

IV, trial arm receiving IFN‐*α*‐2b 15–20 MIU/m^2^ IV daily 5 days per week for 4 weeks; IV and SC, trial arm receiving IFN‐*α*‐2b 15–20 MIU/m^2^ IV daily 5 days per week for 4 weeks followed by IFN‐*α*‐2b 9–10 MIU/m^2^ administered three times per week for 48 weeks.

**Figure 2:**
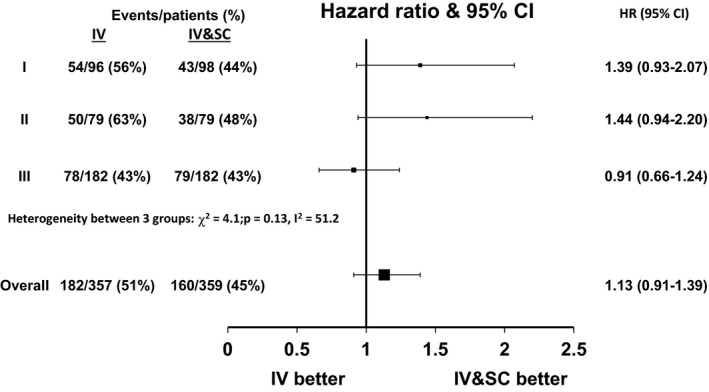
Hazard ratio plot of the treatment effect for overall survival.

The treatment effect estimate for OS favored the longer IV and SC schedule in two of the studies (Table 2). There was some heterogeneity between the cohorts in terms of OS (*χ* ^2^ = 4.1, *P* = 0.13, *I* ^2^ = 51.2, Fig. 2). The overall HR of death was 1.13 for the IV arm compared to the IV and SC arm (95% CI 0.91–1.39, Fig. 2). There were no significant treatment–covariate interactions for OS (Table 3). In a multivariable one‐stage fixed effects analysis, stage (III vs. II, HR = 2.21, 95% CI 1.68–2.90, *P* < 0.0001) and Breslow thickness (>4 mm vs. ≤4 mm, HR = 1.32, 95% CI 1.12–1.56, *P* = 0.001) were significant independent predictors of OS (Table 4).

## Discussion

Patients with stage IIB–IIIC resected melanoma are at high risk of relapse and death. The need for an effective and tolerable adjuvant therapy is paramount. Despite recent advances in melanoma, IFN‐*α* is the only therapy currently licensed for the adjuvant treatment of melanoma, with documented success in improving RFS and, to a lesser extent, OS 3. There is as yet no universally accepted method of administering IFN‐*α* with wide variations in dose, duration, and intensity of treatment reported in the literature 3.

For high‐risk patients who are candidates for adjuvant therapy, a commonly accepted regimen consists of a 4‐week induction phase of IFN‐*α*‐2b 20 MIU/m^2^ administered IV 5 days per week for a total of 4 weeks, followed by 48 weeks of maintenance therapy with IFN‐*α*‐2b 10 MIU/m^2^ per day SC administered three times per week.

In a phase II pilot study we published recently 8, 194 patients with high‐risk resected melanoma were randomized to receive either the IV month of HDI described above or the same regimen followed by 48 weeks of maintenance treatment. We found that OS favored the longer treatment arm. As the study population consisted of a majority of stage IIIB/C patients, this result was consistent with a longer duration of treatment being of greater benefit in a higher risk population.

We have combined these data in a meta‐analysis, together with data from two similar adjuvant HDI studies published previously. All three studies addressed the same question: whether the use of SC interferon for 11 months after a month of higher dose IV administration reduced the risk of melanoma relapse. Although the Greek and Chinese trials used lower starting doses of IFN‐*α*‐2b the greatly reduced frequency of dose interruptions and/or reductions means that all three trials delivered similar total doses of interferon in both the IV alone and IV and SC arms, supporting the combined analysis of the studies.

Disease stage (*P* < 0.0001), as expected, was a significant predictor of both RFS and OS. Breslow thickness, which is integral to melanoma staging, was also a significant predictor of OS (*P* = 0.001) and had a borderline effect for RFS (*P* = 0.02). We also identified a poorer prognosis for acral disease (*P* < 0.0001) for RFS compared with other sites, although this was not predictive of OS (acral vs. other *P* = 0.77).

Our analysis was unable to determine that the 4‐week IV induction phase was noninferior at the 10% level for RFS (*P* = 0.17) compared with induction followed by 11 months maintenance therapy. We analyzed results from 716 patients with high‐risk resected melanoma, whereas a definitive assessment of noninferiority that would be likely to change clinical practice would require over 3000 patients with a 5% alpha level and 90% power, assuming a 50% RFS at 2 years in the standard arm, a recruitment period of 6 years, an additional 2 years of follow‐up, and defining noninferiority as no worse than 3%. Nearly all of the benefit of interferon in terms of prolonged RFS, seen in the Mocellin meta‐analysis 3, has been lost when using only the IV month of HDI (HR = 1.19, 95% CI 0.98–1.45 in multivariate model). This is consistent with the results of the Intergroup E1697 study, designed to compare 4 weeks of adjuvant HDI with observation in patients with intermediate or high‐risk resected melanoma. The trial recruited over 1000 patients (19% with nodal disease), but closed prematurely when interim results showed no difference between the treatment and observation arms 15.

Although there was no significant evidence for heterogeneity of the treatment effect across the three trials, it is interesting that larger HRs for the month of IV interferon were observed for both RFS and OS within the two studies with the worst prognosis populations. This is in agreement with the stratified analysis within the Chinese study, which showed that median RFS was significantly improved for the stage IIIB–IIIC subset of patients receiving the year long HDI regimen (*P* = 0.02). The Chinese cohort consisted entirely of patients with acral melanoma, whereas 80% of the U.K. group had stage IIIB or IIIC disease.

Despite analyzing individual results from over 700 patients, we have been unable definitively to resolve the issue of whether a month of IV interferon is adequate adjuvant treatment for high or intermediate risk melanoma. This is not altogether surprising, given that the benefits of interferon, such as they are, have only really become discernible through meta‐analyses involving several thousand patients. That being said our results cast doubt on the contention that a month of IV HDI is equivalent to a full year of interferon, with trends consistently favoring the longer course of treatment. Taken together with results from E1697, the available evidence suggests that where adjuvant HDI is being considered there is no reason to deviate from the year long regimen described in the ECOG and Intergroup studies.

The effect of ipilimumab, at the 10 mg/kg dose, on relapse‐free survival in the adjuvant setting has recently been reported 16. Risk of relapse was significantly reduced with HR estimate 0.75, compared with 0.82 and 0.74 reported for interferon and HDI in meta‐analyses of observation controlled studies 3, 17. The impact on OS is not yet known and toxicity was substantial, with nearly 50% of patients stopping ipilimumab because of treatment‐related adverse events. Trials with PD‐1 targeted agents are now under way and the relative merits of these, ipilimumab, and interferon will depend upon the tolerability of regimens. In making this assessment, our data do not provide justification for shorter, less toxic interferon treatment.

## Conflict of Interest

None declared.
